# Exploring the impact of inflammatory endotypes on olfactory function and quality of life in chronic rhinosinusitis patients

**DOI:** 10.1016/j.bjorl.2023.101364

**Published:** 2023-11-20

**Authors:** Nájla Nonis Zucoloto, Felipe Silva de Aguiar, Natália Medeiros Dias Lopes, Ellen Cristine Duarte Garcia, Fabrizio Ricci Romano, Thiago Freire Pinto Bezerra, Wilma Terezinha Anselmo Lima, Marco Aurélio Fornazieri

**Affiliations:** a Universidade Estadual de Londrina (UEL), Departamento de Cirurgia Clínica, Londrina, PR, Brazil; b GEM ‒ Centro de Excelência em Pesquisa, Ensino e Atenção à Saúde, Londrina, PR, Brazil; c Universidade de São Paulo, Departamento de Otorrinolaringologia, Ribeirão Preto, SP, Brazil; d Federal Universidade de Pernambuco (UFPE), Departamento de Cirurgia, Divisão de Otorrinolaringologia, Recife, PE, Brazil; e Pontifícia Universidade Católica do Paraná, Departamento de Medicina, Londrina, PR, Brazil; f University of Pennsylvania, Perelman School of Medicine, Smell and Taste Center, Department of Otorhinolaryngology: Head and Neck Surgery, PA, United States

**Keywords:** Rhinosinusitis, Olfaction, Quality of life, Endotype

## Abstract

**Objectives:**

The aim of this observational cross-sectional study was to determine the endotypic inflammatory pattern of a sample of patients with CRS in Brazil, correlate it with olfactory function, and evaluate the clinical severity of the disease.

**Methods:**

In this cross-sectional study, 73 CRS patients were recruited. Patients were classified into type 2 and non-type 2 endotypic patterns based on IgE and eosinophilia levels. All subjects performed the University of Pennsylvania Smell Identification Test (UPSIT®) and responded to the Sino-Nasal Outcome Test (SNOT-22).

**Results:**

The majority of patients had type 2 CRS (*n* = 57, 78.1%). Patients with type 2 CRS compared to non-type 2 CRS had a higher prevalence of nasal polyps (93% vs. 12.5%), asthma (40.3% vs. 12.5%), and non-steroidal anti-inflammatory drug exacerbated respiratory disease (NERD, 17.5% vs. 0%). Type 2 patients also had significantly lower UPSIT® and higher SNOT-22, Lund-Kennedy, and Lund-Mackay scores.

**Conclusion:**

Our study provides evidence that type 2 CRS is associated with a higher prevalence of nasal polyps, asthma, and NERD, as well as decreased olfactory function and worse quality of life scores. These data will contribute to the body of knowledge on CRS and the development of treatments for this disease in Brazil.

**Level of evidence:**

3.

## Highlights

• Type 2 Chronic Rhinosinusitis (CRS) had more severe disease.
• Type 2 CRS had worse olfactory function.
• Type 2 CRS had lower quality of life.
• Type 2 CRS presented higher Lund-Mackay and Lund-Kennedy scores than non-type 2.
• Furthermore, type 2 CRS had a higher prevalence of nasal polyps, asthma, and NERD.

## Introduction

The EPOS 2020 steering committee has taken a comprehensive approach to categorizing Chronic Rhinosinusitis (CRS).^1^ They classify it into primary and secondary forms and further subdivide each into localized or diffuse types based on anatomical distribution. In primary CRS, they assess it by endotype dominance (type 2 or non-type 2) and distinguish clinically localized primary CRS into Allergic Fungal Rhinosinusitis (AFRS) or isolated sinusitis. Diffuse primary CRS is categorized into eosinophilic CRS and non-eosinophilic CRS based on histological eosinophilic cell counts. For secondary CRS, it is again categorized as localized or diffuse and analyzed based on local pathology, mechanical factors, inflammatory factors, and immunological factors, encompassing various clinical phenotypes.

CRS was traditionally categorized into Chronic Rhinosinusitis with Nasal Polyposis (CRSwNP) and Chronic Rhinosinusitis without Nasal Polyposis (CRSsNP) based on phenotype presentations, recent studies have shown that immunological differences and inflammation are crucial even within these primary phenotypes.^2^ Notably, no studies have been conducted in Latin America on this topic.

One groundbreaking study was conducted in 2016 by Tomassen et al. to identify inflammatory endotypes and develop a classification of the disease based on molecular differences, rather than phenotype alone.^3^ The study identified ten subtypes of patients based on the prevalence of several biomarkers, including IFN-gamma, IL-5, IL-17A, IL-22, TNF-alpha, IL-1B, IL-6, IL-8, MPO, ECP, total IgE, specific IgE for S. aureus superantigens, albumin, and TGF-beta 1. The subtypes were then correlated with clinical characteristics, mainly regarding nasal polyposis and asthma. The results showed that the presence of IL-5 was related to a greater presence and severity of nasal polyposis and asthma.

In 2017, Divekar et al. selected other biomarkers to endotypically classify patients with CRS using a cytokine-chemokine immunoassay in samples from 32 patients (26 with CRS and six controls).^4^ Based on the results, they suggested fewer inflammatory patterns than the previous study and placed this feature as an advantage for greater clinical applicability. The results exposed three phenotypic groups and three groups of inflammatory markers, adding to the clinical correlation between these groups. Based on this study, CRS was placed on three pillars: CRS without nasal polyposis and with low levels of asthma, associated with an inflammatory response rich in chemokines and growth factors; CRS with a high prevalence of asthmatics and 100% of nasal polyposis, associated with a type 2 inflammatory response; and CRS with 62% of cases without nasal polyposis, associated with mixed Th1/Th17 response and chemokines and growth factors.^4^

However, there is still a need for further studies focusing on tissue endotyping and the association of inflammatory profiles with clinical presentation, such as olfactory function, to develop more specific and effective therapies. Therapies for CRS should be guided not only by the phenotypic configuration of CRS but mainly by the study of its inflammatory components, which are the pathophysiological basis of the disease. Therefore, this study aims to determine the endotypic inflammatory pattern of a sample of patients with CRS in Brazil, correlate it with olfactory function, and evaluate the clinical severity of the disease.

## Methods

### Population

This is an observational cross-sectional study carried out in a tertiary hospital from March 2020 to December 2021. The convenience sample of this study consisted of patients from Londrina, Brazil. A total of 73 patients aged 14 years or older with primary chronic rhinosinusitis (with or without nasal polyposis) were diagnosed clinically and by nasal endoscopy and computed tomography, following the criteria established by the EPOS 2020 (European Position Paper on Rhinosinusitis and Nasal Polyps).^1^ Exclusion criteria were use of corticosteroids (systemic or topical nasal), antileukotrienes or antimicrobials in the 30 days prior to inclusion in the study, history of an acute exacerbation of rhinosinusitis or airway infection in the 30 days preceding material collection, autoimmune disease, cystic fibrosis, immunodeficiency, ciliary dyskinesia, and unilateral polyps. To define the exclusion criteria, drugs and comorbidities that may alter the inflammatory response or influence the diagnosis of primary CRS were considered.

All patients were instructed and signed the informed consent. Approval was obtained from the Research Ethics Committee (CEP) involving human beings at the Universidade Estadual de Londrina (UEL), attested by certificate number 31203920.3.0000.5231.

### Data collection

Olfactory function was tested by the University of Pennsylvania Smell Identification Test (UPSIT®). The test consists of identifying 40 different odors by the patient and, based on the score obtained (number of correct answers), the olfactory function can be classified into normosmia, hyposmia (mild, moderate, and severe), and anosmia.^5^ The assessment of the quality of life was carried out through the application of the SNOT-22, a questionnaire composed of 22 questions, which address specific questions about the sinonasal disease with general health issues (score from 0 to 110).^6^, ^7^ All patients underwent nasal endoscopy and computed tomography, and classified according to Lund-Kennedy and Lund-Mackay scores, respectively.^8^, ^9^ Finally, individuals were also questioned during the visit regarding demographic data, smoking, alcohol consumption, and previous pathological history, such as asthma, atopy, and drug intolerance.

### Classification

Patients were classified as having type 2 or non-type 2 inflammatory responses. Patients underwent measurement of total blood IgE, serum, and tissue eosinophilia. Type 2 pattern was defined when tissue eosinophilia was ≥10 cells/high power field, serum eosinophilia was ≥250 cells/mcL, or total serum IgE was ≥100 IU/mL.

### Statistical analysis

Patients in both groups had their UPSIT® and SNOT-22 scores submitted to the normality test and compared by the *t*-test for unpaired data. Data obtained during the medical appointment, such as information on smoking, asthma, drug allergy, and atopy, were tabulated and analyzed using the Chi-Square test, with Yates’ continuity correction, when necessary, to assess the association of these variables in each of the two groups. The level of significance adopted in this study was 0.05.

## Results

Table 1 shows that our study comprised 73 patients diagnosed with CRS, including 32 men (43.8%) and 41 women (56.2%). Most patients had type 2 CRS (*n* = 57, 78.1%), which was associated with a higher prevalence of nasal polyps and asthma. Additionally, type 2 individuals presented with decreased olfactory function and worse quality of life scores compared to non-type 2 subjects. As expected, only type 2 patients reported respiratory distress exacerbation to Non-Steroidal Anti-Inflammatory Drugs (NSAID).

**Table 1:** Comparison of demographic and clinical characteristics of the type 2 and non-type 2 chronic rhinosinusitis patients. Significant p values are bolded.

	Total	Type 2	Non-type 2	*p*-value
	(*n* = 73)	(*n* = 57)	(*n* = 16)	
Age, mean (SD)	45.6 (16.2)	45.6 (16.2)	45.6 (16.8)	0.99
Sex, *n* (%)				0.26
Female	41 (56.2)	34 (59.6)	7 (43.8)	
Male	32 (43.8)	23 (40.4)	9 (56.2)	
Smoking, *n* (%)				0.26
Yes	9 (12.3)	5 (8.7)	4 (25)	
No	64 (87.7)	52 (91.3)	12 (75)	
Nasal polyps, *n* (%)				**<0.001**
Yes	55 (75.3)	53 (93)	2 (12.5)	
No	18 (24.7)	4 (7)	14 (87.5)	
Asthma, *n* (%)				**0.03**
Yes	25 (34.3)	23 (40.3)	2 (12.5)	
No	48 (65.7)	34 (59.7)	14 (87.5)	
NERD, *n* (%)				0.11
Yes	10 (13.7)	10 (17.5)	0	
No	63 (86.3)	47 (82.5)	16 (100)	
UPSIT®, median (IQR)	22 (14–28)	20 (12–19.4)	29.5 (26.8–30.3)	**<0.001**
SNOT-22, mean (SD)	50.5 (24.2)	53.8 (25.2)	38.8 (16.1)	**<0.001**

NERD, Non-steroidal anti-inflammatory drug Exacerbated Respiratory Disease; UPSIT, University of Pennsylvania Smell Identification Test; SNOT-22, Sino-Nasal Outcome Test.

Our results demonstrate that a high proportion of patients in the type 2 group exhibited nasal polyps (93%), with a significant proportion also presenting with asthma (40.3%) and non-steroidal anti-inflammatory drug Exacerbated Respiratory Disease (NERD) (17.5%). Conversely, the non-type 2 group had a markedly lower prevalence of nasal polyps (12.5%) and asthma (12.5%).

**Figure 1:**
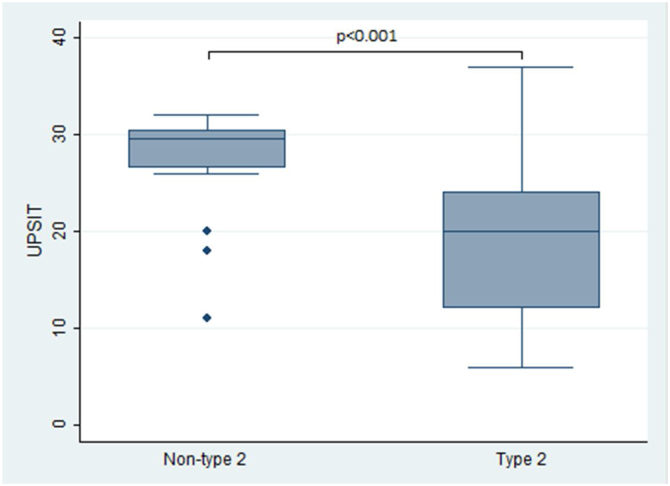
University of Pennsylvania Identification Test (UPSIT®) score comparison between type 2 and non-type 2 chronic rhinosinusitis.

In terms of the primary outcomes of this study, namely the UPSIT® and SNOT-22 scores, the results indicated that patients with type 2 CRS experienced significantly worse outcomes compared to those without type 2 CRS. Specifically, the median UPSIT® score for patients in the type 2 group was 20, which was substantially lower than the median score of 29.5 observed in the non-type 2 group (as illustrated in Fig. 1). Additionally, the mean SNOT-22 score for patients in the type 2 group was 53.8 (SD = 25.2), whereas those in the non-type 2 group had a significantly lower mean score of 38.8 (SD = 16.1).

**Figure 2:**
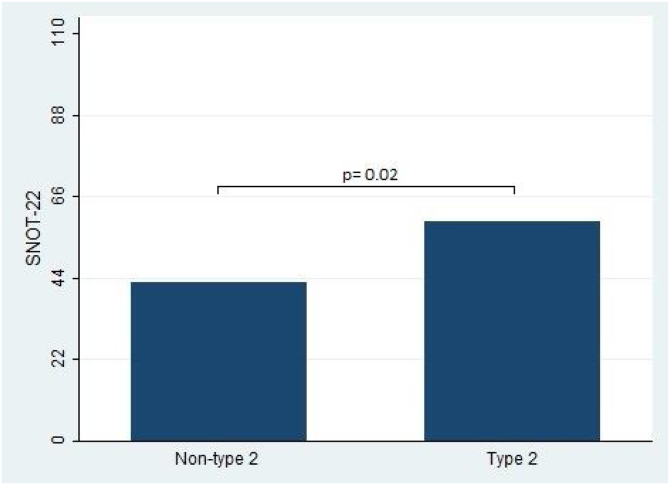
Sino-Nasal Outcome Test (SNOT-22) score comparison between type 2 and non-type 2 chronic rhinosinusitis.

**Figure 3:**
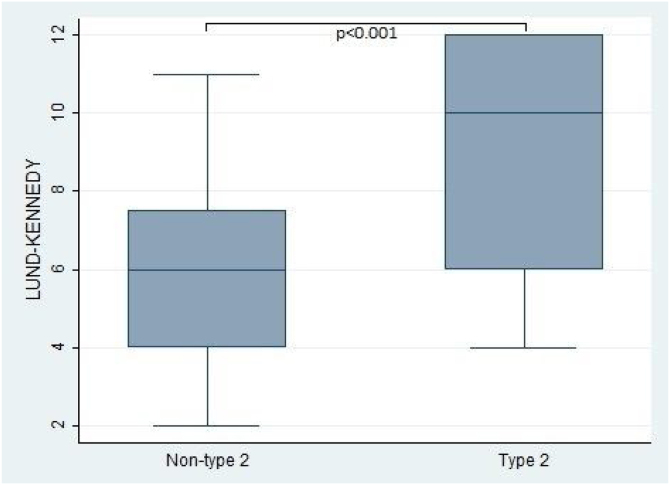
This figure compares the Lund-Kennedy score between patients with type 2 and non-type 2 Chronic Rhinosinusitis. The results demonstrate that patients with type 2 inflammation exhibited significantly higher scores on the Lund-Kennedy scale compared to those without type 2 inflammation.

**Figure 4:**
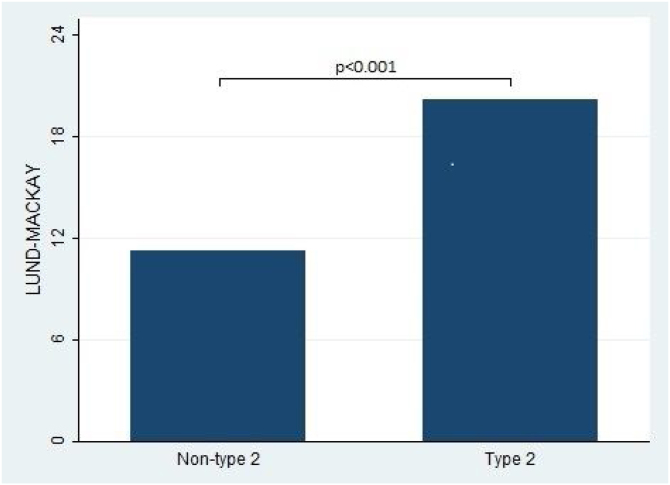
This figure compares the Lund-Mackay score between patients with type 2 and non-type 2 Chronic Rhinosinusitis. The results demonstrate that patients with type 2 inflammation exhibited significantly higher scores on the Lund-Mackay scale compared to those without type 2 inflammation.

Furthermore, the present study observed significant differences in the Lund-Kennedy and Lund-Mackay scores between the type 2 and non-type 2 groups (ps < 0.001, as depicted in Fig. 2). Specifically, the median Lund-Kennedy score for patients in the type 2 group was 10 (IQR: 6–12), which was higher than the median score of 6 (IQR: 4–7.5) observed in the non-type 2 group. Moreover, the mean Lund-Mackay score for patients in the type 2 group was 16.8 (SD = 4.1), which was significantly higher than the mean score of 9.3 (SD = 4.5) observed in the non-type 2 group (Figure 3, Figure 4).

## Discussion

This study provides new data on the prevalence of endotypic patterns in a Brazilian population and their associations with clinical features. We investigated the prevalence of endotypes in 73 Brazilian patients with Chronic Rhinosinusitis (CRS) and their associations with clinical features such as nasal polyps, asthma, Non-steroidal anti-inflammatory drug Exacerbated Respiratory Disease (NERD), olfactory function, and quality of life. Type 2 CRS was the most common endotype (78.1%), and patients with type 2 CRS had a higher prevalence of nasal polyps, asthma, and NERD, as well as worse olfactory function and quality of life compared to non-type 2 CRS patients. The study also found significant differences in the Lund-Kennedy and Lund-Mackay scores between the two groups, with type 2 CRS patients having higher scores.

We revealed a prevalent type 2 inflammatory response pattern within most of the population, which is associated with more severe clinical conditions. Furthermore, this study has established a link between this type of response and a lower quality of life, as well as increased social impairment among affected patients. Hence, there is a pressing need to advocate on guidelines for the use of drugs that act on this subtype of CRS, as immunobiologicals and other medications to be developed, given their significant potential for improving the well-being of affected individuals.^15^

The insights gleaned from this study may harmonize with global research efforts concerning endotypes and their impact on olfactory function in individuals suffering from CRS. Brazil, as a vast and culturally diverse nation, adds a unique dimension to this area of research, which has been relatively underexplored within its borders. Consequently, these findings have the potential to enrich the global body of knowledge and contribute significantly to the development of strategic treatments for CRS. Although the study sample size may not be extensive, these findings shed light on the epidemiological landscape of CRS in Brazil.

Generally, type 2 CRS have higher Lund-Mackay and Lund-Kennedy scores, indicating more severe disease.^1^, ^2^ This may be due to the role of type 2 inflammation in promoting tissue remodeling, fibrosis, and the formation of nasal polyps.^2^ Additionally, type 2 inflammation is associated with increased levels of cytokines and chemokines that promote eosinophilic inflammation, which has been linked to disease severity in CRS.^1^, ^2^, ^3^, ^10^

The association between type 2 inflammation and more severe sinonasal disease has been well established in the literature.^1^ Our finding that patients with type 2 CRS had worse SNOT-22 scores compared to those without type 2 CRS is in line with these previous studies.

We also demonstrated the association between type 2 CRS and poorer olfaction, our results showing a significantly lower UPSIT® score in the type 2 group compared to the non-type 2 group. The presence of eosinophilic inflammation in type 2 CRS can lead to damage of the olfactory sensory neurons, resulting in reduced olfactory function. This is consistent with previous studies that have demonstrated a link between type 2 inflammation and olfactory dysfunction in CRS.^11^, ^12^ Additionally, our study found that type 2 CRS patients had a higher prevalence of nasal polyps, which can further contribute to olfactory dysfunction by obstructing airflow to the olfactory epithelium.

The present study did not find a statistically significant difference in gender distribution between type 2 and non-type 2 CRS patients, although there were more men in the non-type 2 group and the majority in type 2 were women. This finding is not consistent with some previous studies, which have reported a male predominance in CRS with nasal polyps, more common in type 2 CRS.^13^, ^14^

The study has several strengths that enhance its validity and reliability. The population was well-defined and identified based on the criteria established by EPOS 2020, which are widely accepted in the field. Seventy-three patients were enrolled, which adds statistical power to the study. The study employed validated tools (UPSIT® and SNOT-22) for data collection, which increases the accuracy of the results. However, the study has some weaknesses that need to be addressed. First, the study used a convenience sample, which may limit the generalizability of the results. Second, the study is cross-sectional in nature, which means that causality cannot be inferred. Finally, the study did not provide information on the intra- and inter-rater reliability of the tools used, which could have affected the validity of the results.

## Conclusion

This study provides new insights into the prevalence and clinical features associated with endotypes in Brazilian patients with chronic rhinosinusitis. Type 2 CRS was the most common endotype, and patients with this endotype had more severe disease, including higher Lund-Mackay and Lund-Kennedy scores, worse olfactory function, and lower quality of life. The study also found a higher prevalence of nasal polyps, asthma, and NERD in patients with type 2 CRS. Our findings are consistent with previous studies, which have demonstrated the association between type 2 inflammation and more severe sinonasal disease. Further research is needed to confirm the potential association between smoking and non-type 2 CRS observed in this study. Overall, these results may have implications for the development of personalized treatments for patients with chronic rhinosinusitis based on their endotype.

## Authors’ contributions

The decision to include eight authors in this article was guided by the extensive contributions of each member of the research team in collecting clinical and laboratory data, writing the article or giving precious inputs for better results and conclusions. We recognize that data collection is a fundamental step in scientific research, and the collaborative approach was essential to ensure the accuracy and reliability of our results.

The authors of this article were involved in data collection from the beginning of the study. Each author contributed in a unique way to the definition of collection protocols, obtaining samples, performing laboratory analyzes and rigorously documenting clinical information. This equitable distribution of responsibilities resulted in a wealth of data covering different aspects of our investigation.

## Funding

None.

## Conflicts of interest

The authors declare no conflicts of interest.
